# Case report: A case report of co-morbidity of cervical intraepithelial neoplasia III and urethral cancer associated with HPV16

**DOI:** 10.3389/fonc.2024.1423874

**Published:** 2024-07-09

**Authors:** San Zhu, Yuhao Liu, Ce Bian, Yan Luo, Manman Zhu, Lingyun Yang

**Affiliations:** ^1^ Department of Gynecology and Obstetrics, West China Second University Hospital, Sichuan University, Chengdu, China; ^2^ Key Laboratory of Birth Defects and Related Diseases of Women and Children, Ministry of Education, Sichuan University, Chengdu, China; ^3^ Department of Urology, West China Fourth Hospital, Sichuan University, Chengdu, China

**Keywords:** cervical intraepithelial neoplasia III, urethral squamous cell carcinoma, lymph nodes metastasis, human papillomavirus, cancer diagnosis

## Abstract

In this report, we present a case of a woman with concurrent cervical intraepithelial neoplasia grade III (CIN III) and urethral cancer, both associated with HPV16 infection. This unique case was initially brought to attention due to postmenopausal vaginal bleeding, despite the absence of urological symptoms and negative tumor markers. An unexpected discovery of pelvic lymph node metastasis during a hysterectomy intended for CIN III highlighted the rare coexistence of these conditions, with urethral cancer also linked to HPV-16 within the urethral lesion. This case emphasizes the diagnostic challenges faced by HPV-related cervical lesions and the critical need for increased vigilance, even when urological symptoms are not apparent. The findings underline the potential complexity of HPV-associated lesions and advocate for comprehensive screening strategies to ensure the timely detection and management of such intricate cases.

## 1 Introduction

Cervical Intraepithelial Neoplasia III (CIN-III), characterized by severe epithelial dysplasia, is a critical precursor to cervical cancer, typically indicative of a condition not accompanied by lymph node metastasis. The standard diagnostic approach for CIN-III predominantly relies on colposcopic-guided biopsy. Imaging techniques like Computed Tomography (CT), Magnetic Resonance Imaging (MRI), or Positron Emission Tomography-Computed Tomography (PET-CT) are not routinely used in the initial evaluation of CIN-III due to their limited role in assessing superficial lesions. However, they can be instrumental in advanced stages or cases with suspected metastasis (1). It is predominantly associated with high-risk human papillomavirus (HPV) infections, especially types HPV-16 and HPV-18 (2). HPV’s role extends beyond the cervix, affecting various epithelial tissues, including the skin, oral cavity, and genital mucosa (3). Primary urethral cancer (UC) is a rare malignancy accounting for less than 1% of all tumors. Primary urethral squamous cell carcinoma(USCC) represents this disease’s most prevalent histological subtype, with a higher incidence observed in males (4).

In our presented case, we highlight the exceptional occurrence of CIN-III with extensive pelvic lymph node metastasis, indicative of HPV-related USCC. Notably, the association between HPV and primary urethral cancer has historical roots, with Kitamura et al. reporting an HPV-16-positive case among bladder tumors as early as 1988 (5). Recent meta-analyses underscore the heightened risk of subsequent primary cancers in the urethra for women surgically treated for CIN-II and CIN-III (SIR 4.05 95% exact CI: 1.10–10.56) (6). The co-occurrence of multiple malignancies within a single individual often hints at a shared etiological factor. Nevertheless, our understanding remains limited due to a paucity of relevant case reports and mechanistic investigations. Understanding the co-occurrence of CIN-III and HPV-related USCC, as in the presented case, offers a unique window into the complex interplay between HPV and multiple malignancies of the genital tract and adjacent areas. This case underscores the importance of vigilance and comprehensive evaluation in patients presenting with HPV-related lesions, given the potential for multisite involvement.

## 2 Case description

A 64-year-old postmenopausal woman was presented with postcoital vaginal bleeding and no dysuria or bladder irritation symptoms. The patient had no significant medical history, including regular health check-ups, hormone therapy, smoking, or a known familial history of malignancies. A comprehensive gynecological examination revealed no visible lesions externally. However, her cervical screening indicated HPV-16 positivity, a high-risk oncogenic strain. The HPV viral load, measured using the surrogate relative light units/control from Hybrid Capture 2 (HC2), was 1220.90 RLU/CO (with 1 RLU/CO as the threshold).

**Figure 1:**
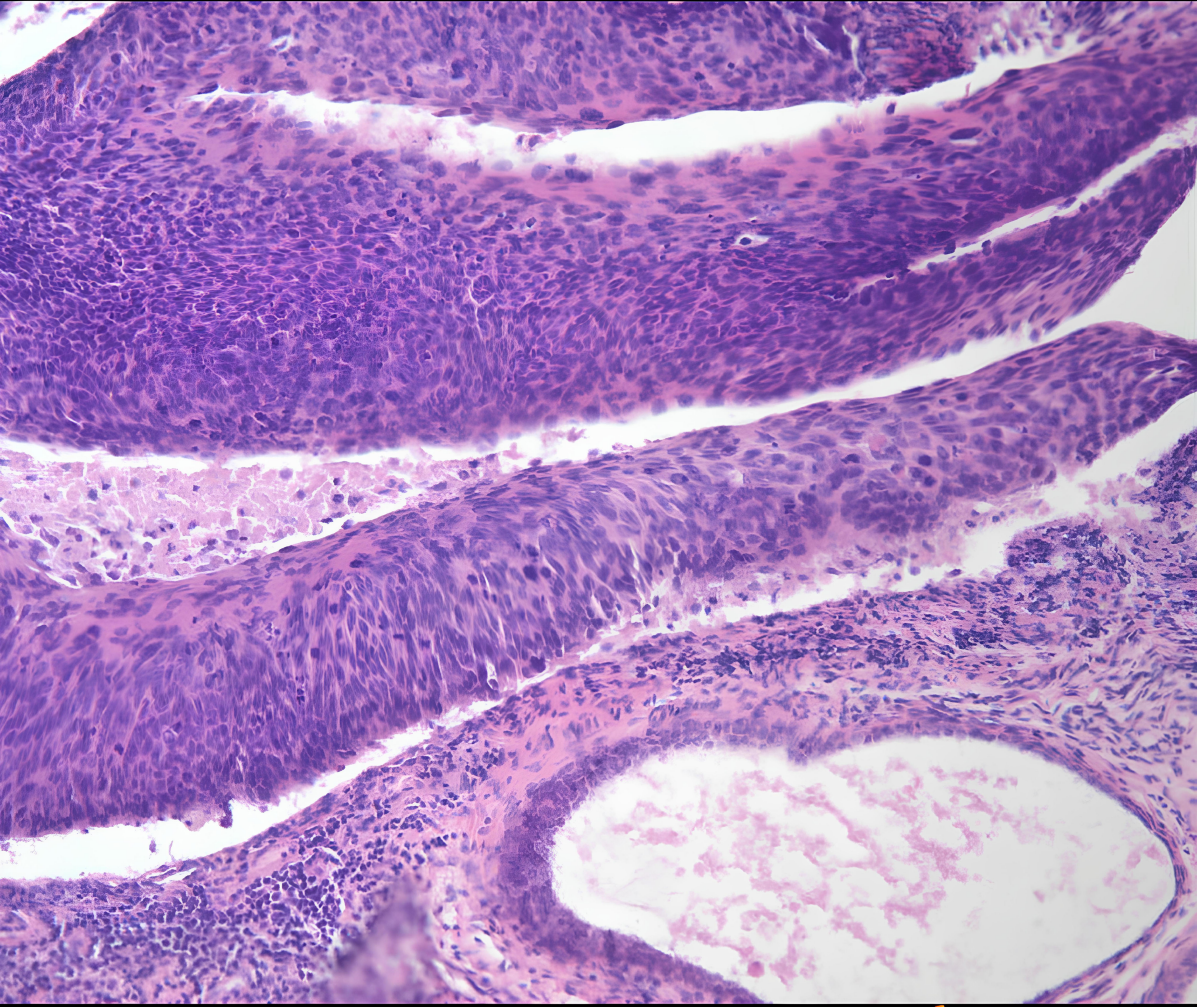
The pathological examination of cervical tissue biopsy revealed chronic inflammation associated with erosion and metaplasia, multifocal CIN-III lesions, and glandular involvement.

Subsequent liquid-based cytologic testing identified a high-grade squamous intraepithelial lesion (HSIL), further confirmed as CIN-III through a colposcopy-assisted cervical biopsy (**Figure 1**). Tumor markers (HCG, CEA, AFP, CA125, CA19-9, CA15-3, SCC) yielded negative results.

**Figure 2:**
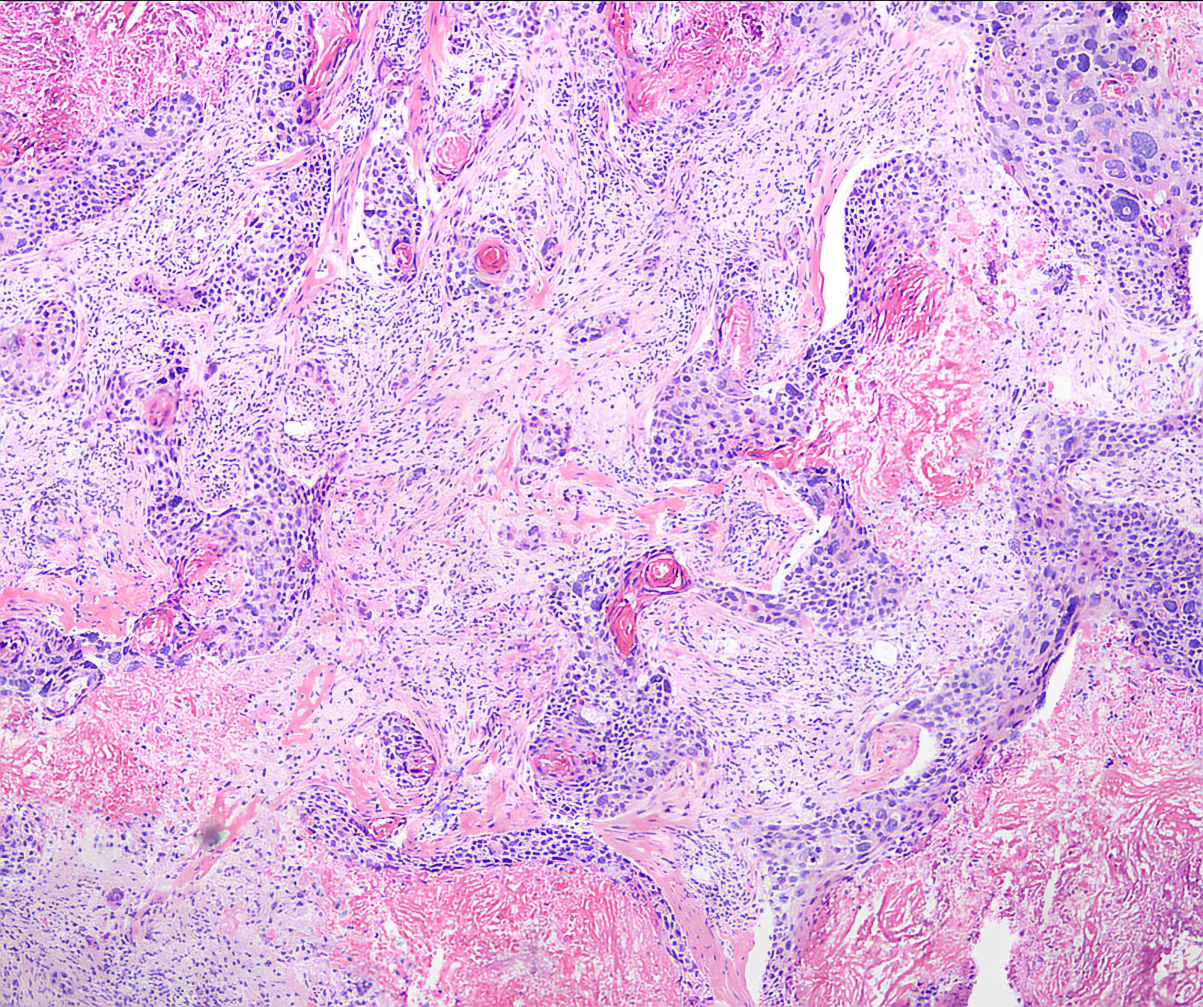
The enlarged pelvic lymph node’s pathological examination detected cancer metastasis.

A conical endocervicectomy was performed initially, followed by a laparoscopic extrafascial hysterectomy and bilateral salpingo-oophorectomy, given the absence of advanced lesions. During the surgical procedure, an enlarged right obturator lymph node, approximately 5x5x4 cm^3^ in size, was detected unexpectedly. It is closely adjacent to the external iliac vein and encircled by the obturator nerve (**Supplementary 1**). Our meticulous en bloc laparoscopic lymphadenectomy procedure allowed for the excision of this node, which, upon histological examination, revealed a moderately differentiated squamous cell carcinoma (**Figure 2**).

Given the lack of conclusive evidence to support a diagnosis of primary cervical cancer, no further surgical intervention was pursued. However, the postoperative pathology and subsequent immunohistochemistry (IHC) tests indicated the presence of cancer metastasis in the lymph node. The urethral tumor was characterized by markers such as P16 (diffuse positive), CK 5/6 (positive), P63 (positive), GATA3 (partially positive), and Uroplakin III (positive). The follow-up PET-CT scan revealed an irregular neoplasm in the middle segment of the urethra, characterized by heterogeneous thickening of the urethral wall, thus confirming the dual diagnosis of CIN-III and urethral cancer. The patient was then referred to the urology department, where she underwent cystoscopy-guided transurethral resection of the urethral neoplasm.

**Figure 3:**
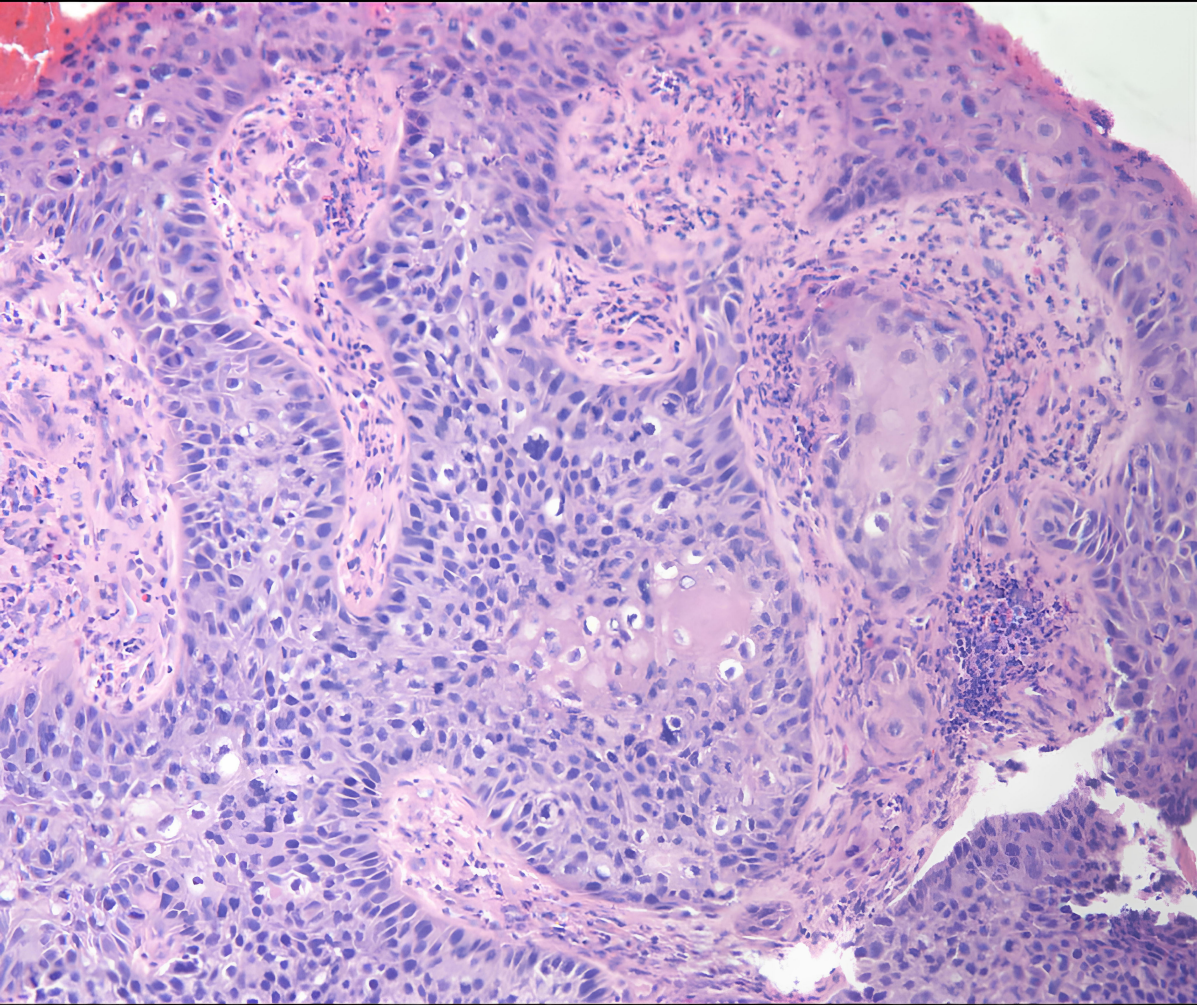
The urethral lesion’s pathological report suggested the presence of moderately differentiated squamous cell carcinoma.

**Figure 4:**
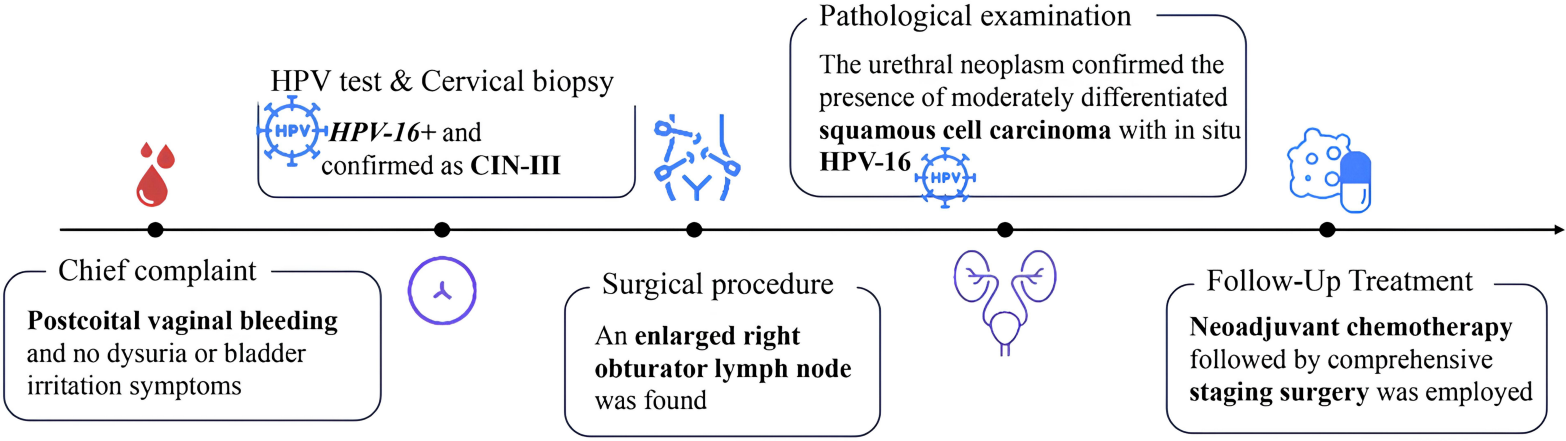
The timeline showcases relevant data from the whole care.

The pathological examination of the resected tissue confirmed the presence of moderately differentiated squamous cell carcinoma, and IHC results revealed the infection with *in-situ* HPV-16 (**Figure 3**). Upon the definitive diagnosis of urethral squamous cell carcinoma, the patient underwent neoadjuvant chemotherapy followed by comprehensive staging surgery. She is currently undergoing regular follow-up (**Figure 4**).

## 3 Discussion

This case presents a rare instance of simultaneous cervical intraepithelial neoplasia III (CIN-III) and urethral squamous cell carcinoma (USCC) in a postmenopausal woman. Initially, she presented with postcoital vaginal bleeding without typical urological symptoms, highlighting the stealthy nature of HPV-related malignancies. In any population, individuals may experience various cancer occurrences. Simultaneous occurrences of two or more cancer types within one individual often result from specific factors, such as susceptibility gene mutations, shared exposures (e.g., radiation, smoking, viral infections), or developmental abnormalities (7).

The presence of HPV-16 in both the cervical and urethral lesions underscores the virus’s oncogenic potential across different anatomical sites. Globally, high-risk HPV types, particularly HPV-16, are implicated in approximately 10% of cancers, including the majority of cervical and significant percentages of anal, vulvar, vaginal, and penile cancers (8). HPV is highly tissue-specific, infecting epithelial cells of the skin, and oral and genital mucosa primarily through sexual activity, although skin-to-skin contact is also a viable transmission mode (3, 9). The high tissue specificity of HPV (3) suggests a plausible route for the virus to cause multiple primary cancers in the genitourinary tract. Due to the anatomical proximity of the urinary and genital tracts, the urethra is particularly susceptible to HPV infection (10, 11). This susceptibility is supported by studies like those of Kitamura et al. and Wiener et al., who identified a strong link between HPV-16 and urethral carcinomas using molecular techniques like Southern blotting and polymerase chain reaction (PCR) (5, 11, 12). A retrospective cohort study reported a four-fold increase in the risk of subsequent primary urethral cancers in women treated for CIN II and CIN III(SIR 4.05 95% exact CI: 1.10–10.56), highlighting the need for vigilant screening protocols in high-risk HPV-positive patients (6). The connection with urethral cancer still remains underexplored, despite the recognized association between HPV and cervical lesions. This oversight became apparent in our case when a metastasis to a pelvic lymph node was discovered, which had been initially overlooked due to the absence of urological symptoms.

The oversight of urethral cancer in our case was critical and thought-provoking, though we had adhered strictly to the treatment protocols for postmenopausal women with abnormal vaginal bleeding as outlined in the guidelines. There is a significant risk that a surgeon tasked with addressing CIN III may not possess the necessary qualifications for specialized cancer surgery, potentially leading to the dissemination of tumor cells during the operation. This recurring cycle of diagnosis and treatment not only escalates the financial and emotional burden on the patient but also increases the strain on healthcare resources. Although the definitive link between HPV and urethral cancer remains elusive, existing studies have indicated a high risk and suggested a potential correlation (5, 6, 10–12). The most recent review and meta-analysis demonstrated a significant association between HPV and urethral cancer (UC), with an odds ratio of 7.84, corroborating another meta-analysis on UC risk (13, 14). A statistically significant difference in the prevalence of HPV infection was observed among different histological subtypes of urethral cancer, particularly in squamous cell carcinoma. Notably, HPV-16 was the most prevalent genotype in UC, reinforcing previous findings that high-risk HPV genotypes, primarily HPV-16, are associated with an increased risk of UC (8, 14, 15). Our findings emphasize the possible mediating role of HPV in these co-occurring conditions.

The close association between HPV infection and both cervical and urological pathologies necessitates increased vigilance in managing patients with HPV-related cervical lesions. It is crucial to consider the possibility of concurrent urological malignancies in patients with HPV-related cervical lesions, especially the high-risk ones like HPV16. As such, imaging modalities such as urinary ultrasound and CT should be considered for comprehensive screening of not only pelvic but also urinary tract malignancies in patients with HPV-related cervical lesions, even if those are only precancerous. The presence of enlarged lymph nodes before surgery should prompt clinicians to conduct further investigations, such as MRI, PET-CT, or even cystoscopy, to rule out USCC or other urinary tract tumors. Precise and timely detection of urethral cancer plays a pivotal role in facilitating tailored therapeutic interventions and enhancing patient prognosis. This significance is underscored by its capacity to refine the assessment of optimal diagnostic and treatment timings, thereby diminishing the necessity for repeated surgical interventions. Furthermore, it aids in accurately evaluating surgical complexity and modality selection, consequently reducing the likelihood of intraoperative contact or injury to occultly afflicted tissues, which may precipitate tumor dissemination in the absence of a definitive diagnosis.

In conclusion, the co-occurrence of CIN-III and USCC in the same patient underscores the need for a holistic approach to HPV-associated lesions, advocating for enhanced screening that includes both pelvic and urinary tract malignancies. Specifically, a comprehensive screening strategy for HPV-related cervical lesions (even if only precancerous), including urinary ultrasound and CT scans for both pelvic and urinary tract malignancies, is recommended. Future research should aim to unravel the mechanisms by which HPV contributes to the development of multiple primary cancers, particularly in the genitourinary tract, and explore the potential benefits of comprehensive screening protocols for asymptomatic high-risk HPV-positive patients. In the near future, the elucidation of characteristics related to HPV-associated cervical lesions coexisting with urethral cancer is poised to pave the way for novel and personalized treatment modalities. The role of prophylactic HPV vaccination in reducing the incidence of such cases also warrants further investigation, especially in populations at higher risk of developing HPV-associated malignancies.

## Compliance with ethics guidelines

Informed Consent: This case was conducted following ethical approval and consent procedures authorized by the Medical Ethical Committee of West China Second University Hospital, Sichuan University, and the study was performed in accordance with the ethical standards as laid down in the 1964 Declaration of Helsinki and its later amendments or comparable ethical standards. Informed consent was obtained from all patients to be included in the study.

## Scope statement

Our manuscript presents a rare case of concurrent cervical intraepithelial neoplasia III (CIN-III) and primary urethral squamous cell carcinoma (USCC), linked to high-risk human papillomavirus (HPV) infection. This case study highlights the significance of comprehensive evaluation in patients with HPV-related lesions, underlining the complex interplay between HPV and multiple malignancies within the genitourinary tract. Our findings emphasize the necessity of screening for urinary tract lesions in instances of cervical precancerous lesions associated with high-risk HPV infections, contributing valuable insights into cancer epidemiology, diagnostics, and management strategies. This work aligns with Frontiers in Oncology’s mission to advance multidisciplinary cancer research, supporting the journal’s aim to bridge the gap between basic research and clinical applications. Additionally, it underscores the importance of innovative screening strategies for early detection and management of cancer. By offering a unique perspective on HPV’s role in genitourinary malignancies, our manuscript advocates for early intervention and underscores the potential for improving patient outcomes, thereby contributing to the future of cancer care.

## Data availability statement

The original contributions presented in the study are included in the article/**Supplementary Material**. Further inquiries can be directed to the corresponding author.

## Ethics statement

The studies involving humans were approved by the Medical Ethical Committee of West China Second University Hospital, Sichuan University. The studies were conducted in accordance with the local legislation and institutional requirements. The human samples used in this study were acquired from primarily isolated as part of your previous study for which ethical approval was obtained. Written informed consent for participation was not required from the participants or the participants’ legal guardians/next of kin in accordance with the national legislation and institutional requirements. Written informed consent was obtained from the individual(s) for the publication of any potentially identifiable images or data included in this article.

## Author contributions

SZ: Data curation, Investigation, Software, Writing – original draft, Writing – review & editing. YHL: Investigation, Resources, Software, Supervision, Writing – review & editing. CB: Conceptualization, Project administration, Supervision, Writing – review & editing. YL: Conceptualization, Investigation, Writing – review & editing. MZ: Methodology, Software, Writing – review & editing. LY: Conceptualization, Investigation, Project administration, Resources, Supervision, Writing – review & editing.

## Supplementary material

Supplementary 1
**(A)** The enlarged lymph node was firmed in texture and located on the medial aspect of the internal iliac artery, closely abutting the vascular wall of the external iliac vein, extending inwards and downwards towards the obturator fossa while encasing the obturator nerve. **(B)** Laparoscopic lymphadenectomy with a precise en bloc technique: Three key anatomical reference points were utilized during pelvic lymph node dissection: the internal iliac artery, the obturator nerve, and the external iliac artery. It was expected to confine the operating field between the internal and external iliac arteries.
