# Supplementary figures and images for: Case report: A case report of co-morbidity of cervical intraepithelial neoplasia III and urethral cancer associated with HPV16
Source: Front Oncol. 2024 Jul 9;14:1423874. doi: 10.3389/fonc.2024.1423874 (PMC11263100; doi:10.3389/fonc.2024.1423874)

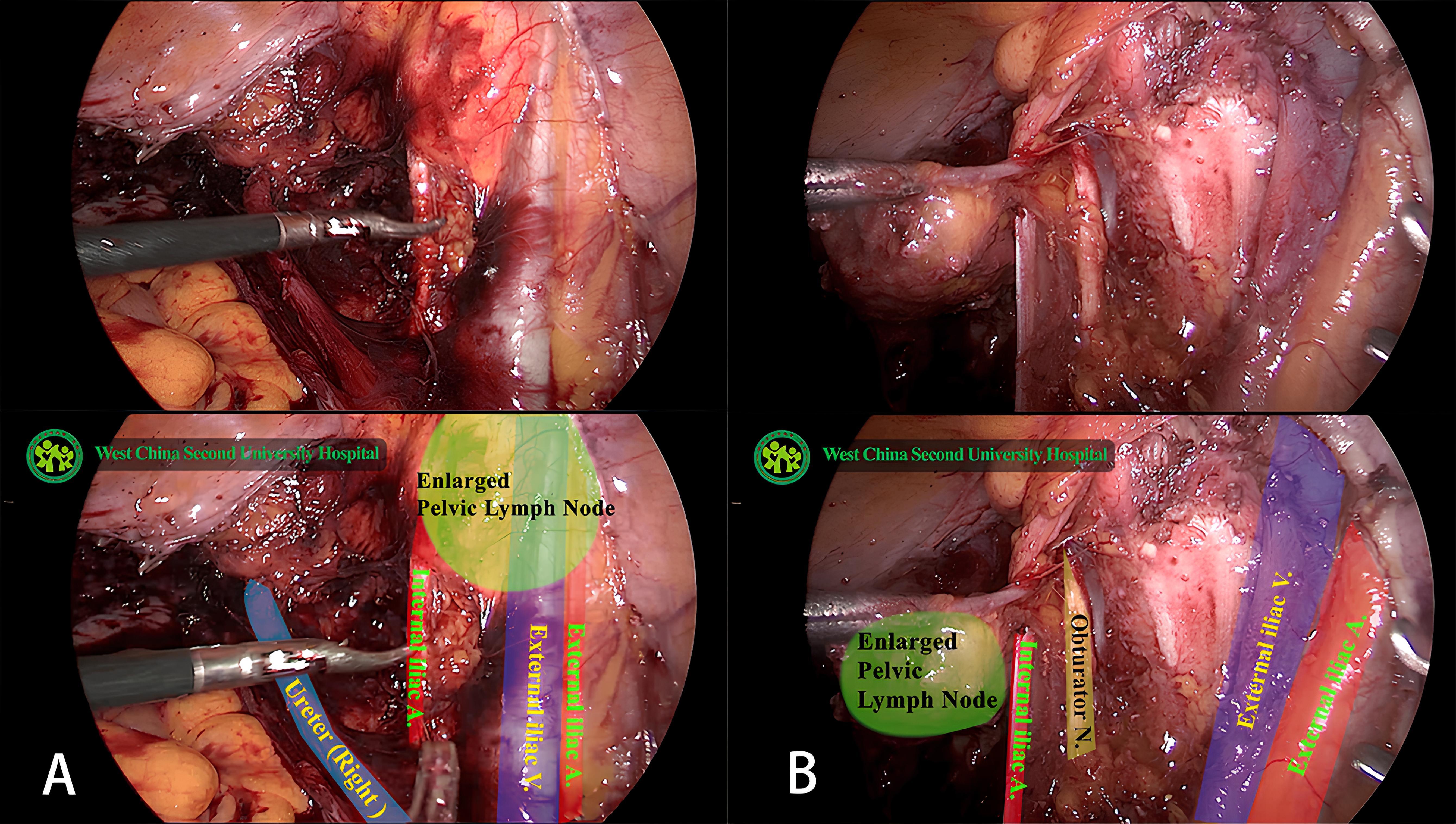

Supplement: Supplementary 1 — (A) The enlarged lymph node was firmed in texture and located on the medial aspect of the internal iliac artery, closely abutting the vascular wall of the external iliac vein, extending inwards and downwards towards the obturator fossa while encasing the obturator nerve. (B) Laparoscopic lymphadenectomy with a precise en bloc technique: Three key anatomical reference points were utilized during pelvic lymph node dissection: the internal iliac artery, the obturator nerve, and the external iliac artery. It was expected to confine the operating field between the internal and external iliac arteries. [file Image_1.jpeg]
